# Supplementary material for: Membrane Curvature Generation by the Caveolin 8S Complex and the Role of Cholesterol
Source: J Chem Theory Comput. 2026 Aug 19;22(17):9167–78. doi: 10.1021/acs.jctc.6c00973 (PMC13564364; doi:10.1021/acs.jctc.6c00973)
Supplement: Supplementary file 1 [file ct6c00973_si_001.pdf]

SUPPORTING INFORMATION

Membrane Curvature Generation by the Caveolin 8S Complex and the Role of Cholesterol

Sayyid Yobhel Vasquez Rodriguez[1] , Themis Lazaridis*[2,3],

1. CCNY Undergraduate Program, Biology Graduate
City College of New York/CUNY, 160 Convent Ave, New York, NY 10031, USA
2. Department of Chemistry and Biochemistry,
City College of New York/CUNY, 160 Convent Ave, New York, NY 10031, USA

3. Graduate Programs in Chemistry, Biochemistry, and Physics,
The Graduate Center, City University of New York,365 Fifth Ave., New York, NY 10016, USA

* Tel. (212) 650-8364 Email: tlazaridis@ccny.cuny.edu

Table S1. Details of the systems run.

	Atoms Total	Lipids Total	Proximal	Distal	Lipid in Barrel	TIP3P	K+/Cl-	Duration (μs)
POPC	2429020	2207	1000	1200	7	702287	1567 / 1534	2
Amber FF POPC	2429020	2207	1000	1200	7	702287	1567 / 1534	2
303 K POPC	2429020	2207	1000	1200	7	702287	1567 / 1534	2
ISO POPC	2429020	2207	1000	1200	7	702287	1567 / 1534	1
POPC Run2	2429020	2207	1000	1200	7	702287	1567 / 1534	2
CONS POPC	2429020	2207	1000	1200	7	702287	1567 / 1534	5
CONS POPC Run2	2429020	2207	1000	1200	7	702287	1567 / 1534	2
POPC/CHOL	2195764	1827 / 790	831 / 360	992 / 426	4 / 4	622131	1403 / 1370	4
POPC/CHOL Run2	2195764	1827 / 790	831 / 360	992 / 426	4 / 4	622131	1403 / 1370	2
POPC/CHOL no barrel lipids	2194932	1823 / 786	831 / 360	992 / 426	N/A	622131	1403 / 1370	2
POPC/CHOL ISO after 4us SEMIISO	2195764	1827 / 790	831 / 360	992 / 426	4 / 4	622131	1403 / 1370	2
9DN0	2508673	2205	1000	1200	5	729273	1571 / 1604	1.93
9DNO ISO Run2	2508673	2205	1000	1200	5	729273	1571 / 1604	1
9DN1	2725938	2205	1000	1200	5	800031	1729 / 1718	2
9DN1 Run2	2725938	2205	1000	1200	5	800031	1729 / 1718	1
POPC/CHOL/PIP (SAPI 33-35)	2166161	1617 / 688 / 85 / 94 / 85	724 / 324 / 41 /46 /38	888 / 363 / 44 / 48 / 47	5 / 1 / 0 / 0 / 0	610178	2971 / 1354	2
POPE/POPG/TOCL2 (E. coli mimic)	2322850	1817 / 501 / 123	839 / 227 / 50	973 / 272 / 73	5 / 2 / 0	658216	2203 / 1423	1.99
POPC/CHOL/POPE/POPS (Mam. Asym)	2056228	1015 / 839 / 585 / 312	115 / 395 / 437 / 312	896 / 442 / 147 / 0	4 / 2 / 1 / 0	572892	2085 / 1302	N/A
Palmitoylated	2447391	2205	1000	1200	5	708146	1581 / 1548	2

Cav3 Alphafold	2768635	2207	1000	1200	7	814174	1806 / 1762	2
Cav1 full length	2935652	2206	1000	1200	6	868134	1994 / 1939	2
Cav3 swissmodel	2482524	2208	1000	1200	8	720200	1531 / 1531	2
Cav2 swissmodel	2098101	2152	1000	1147	5	594938	1256 / 1245	2
POPC membrane only	2402186	2000	1000	1000	N/A	710428	1451 / 1451	2
P132L	2463779	2207	1000	1200	7	713855	1567 / 1534	1.81
S80E	2481273	2206	1000	1200	6	719731	1578 / 1534	1.97
DDM 572	797675	N/A	N/A	N/A	N/A	242220	0 / 665	1cons / 2 uncons
DDM 572 277 K from flat	797675	N/A	N/A	N/A	N/A	242220	0 / 665	2
DDM 572 277 K from conical	797675	N/A	N/A	N/A	N/A	242220	0 / 665	2
DDM 961	757927	N/A	N/A	N/A	N/A	218505	0 / 609	2cons / 2 uncons
Hexane 14148	306313	N/A	N/A	N/A	N/A	0	0 / 0	1.93

Table S2. Spatial extent of the complex (maximum – minimum x,y,z coordinate) after aligning it with the xy plane. Dz shows the extent of conical shape.

	DX	DY	DZ
POPC	137.81 ± 4.43	134.19 ± 3.63	62.62 ± 3.70
Amber FF POPC	136.46 ± 2.51	132.98 ± 1.44	56.40 ± 2.13
303 K POPC	138.81 ± 3.59	135.58 ± 3.37	65.93 ± 3.07
ISO POPC	139.64 ± 4.42	134.53 ± 3.62	63.68 ± 5.08
NR1 POPC	140.00 ± 4.35	132.79 ± 2.94	63.55 ± 2.71
CONS POPC	(constrained,	similar to cryoEM)	
CONS NR1 POPC	135.61 ± 1.32	135.01 ± 1.34	41.57 ± 0.92
POPC/CHOL	141.33 ± 4.78	131.73 ± 2.91	60.89 ± 2.26
POPC/CHOL NR1	139.56 ± 4.82	136.18 ± 3.58	63.13 ± 2.69

POPC/CHOL NO MEM2	137.33 ± 3.28	134.80 ± 4.21	58.85 ± 1.77
POPC/CHOL ISO after 4usSEMIISO	152.30 ± 4.35	131.21 ± 2.40	60.62 ± 1.81
9DN0	132.29 ± 1.23	130.87 ± 1.18	50.56 ± 4.36
9DN0 initial	132.08	131.41	39.98
9DNO ISO (NR1)	133.46 ± 1.74	132.43 ± 1.51	56.61 ± 5.32
9DN1	126.47 ± 1.36	125.67 ± 1.38	64.40 ± 1.71
9DN1 initial	132.44	131.53	53.73
9DN1 NR1	126.81 ± 1.38	125.63 ± 1.43	62.23 ± 2.18
POPC/CHOL/PIP (SAPI 33-35)	137.99 ± 3.63	133.61 ± 2.72	61.61 ± 3.19
POPE/POPG/TOCL2 (E. coli mimic)	147.43 ± 5.85	138.11 ± 4.55	64.98 ± 2.33
POPC/CHOL/POPE/POPS (Mam. Asym)	139.76 ± 3.02	135.75 ± 3.17	59.23 ± 3.25
Palmitoylated	137.38 ± 2.86	136.34 ± 4.03	63.31 ± 3.31
Alphafold	158.44 ± 9.59	160.14 ± 10.37	65.07 ± 3.47
Full-length cav1 (res 49-177)	138.55 ± 3.43	133.96 ± 2.99	62.76 ± 3.15
Cav3	138.65 ± 5.22	132.18 ± 3.30	63.66 ± 3.03
Cav2	136.20 ± 3.47	132.84 ± 3.41	50.19 ± 4.92
POPC membrane only	N/A	N/A	N/A
P132L	138.50 ± 3.91	134.40 ± 4.38	64.59 ± 3.27
S80E	142.64 ± 3.51	138.46 ± 5.32	64.88 ± 2.84
DDM 572_UNCONS	143.23 ± 4.03	138.82 ± 4.23	63.97 ± 2.90
DDM 961_UNCONS	141.58 ± 4.54	135.07 ± 3.74	60.27 ± 2.10
Hexane 14148	(complex distorts,	DZ is misleading)	
DDM 572_flat_start_277K	139.57 ± 2.90	137.01 ± 3.68	55.68 ± 2.19
DDM 572_conical_start_277K	140.98 ± 3.95	137.12 ± 3.60	65.60 ± 1.29

Supporting text

A number of replicate runs and controls were run to ensure the reliability of the results.

a) A pure POPC membrane patch was run without the 8S complex. The patch remained flat for 1.3 μs, and then acquired curvature. The final curvature was R ~19 nm, similar to the POPC-cholesterol system.

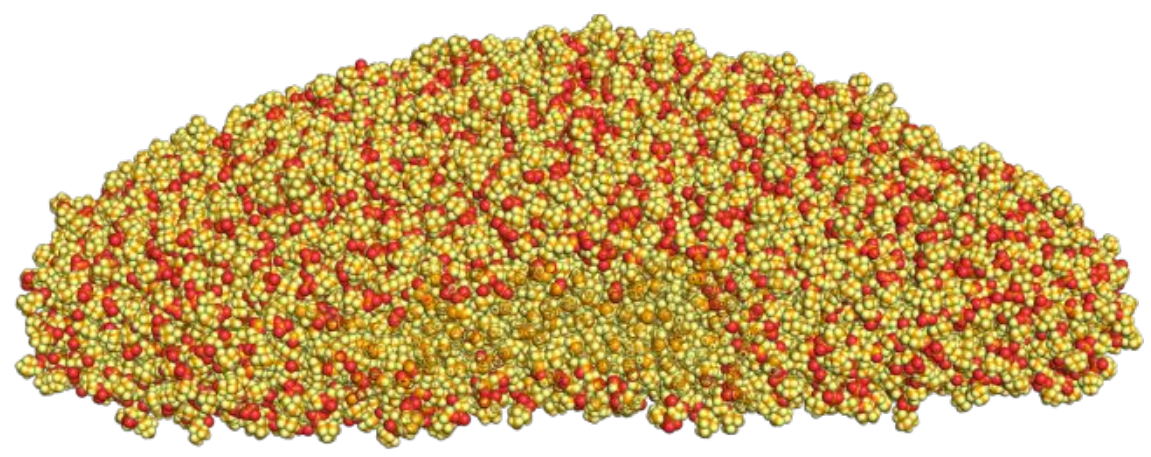

Fig. S1. POPC membrane without protein after 2 μs. The radius of curvature of the outer leaflet is ~ 185 nm.

b) Replicate runs were conducted for the nonvertebrate caveolins, starting from different initial velocities. The replicates gave very similar results.

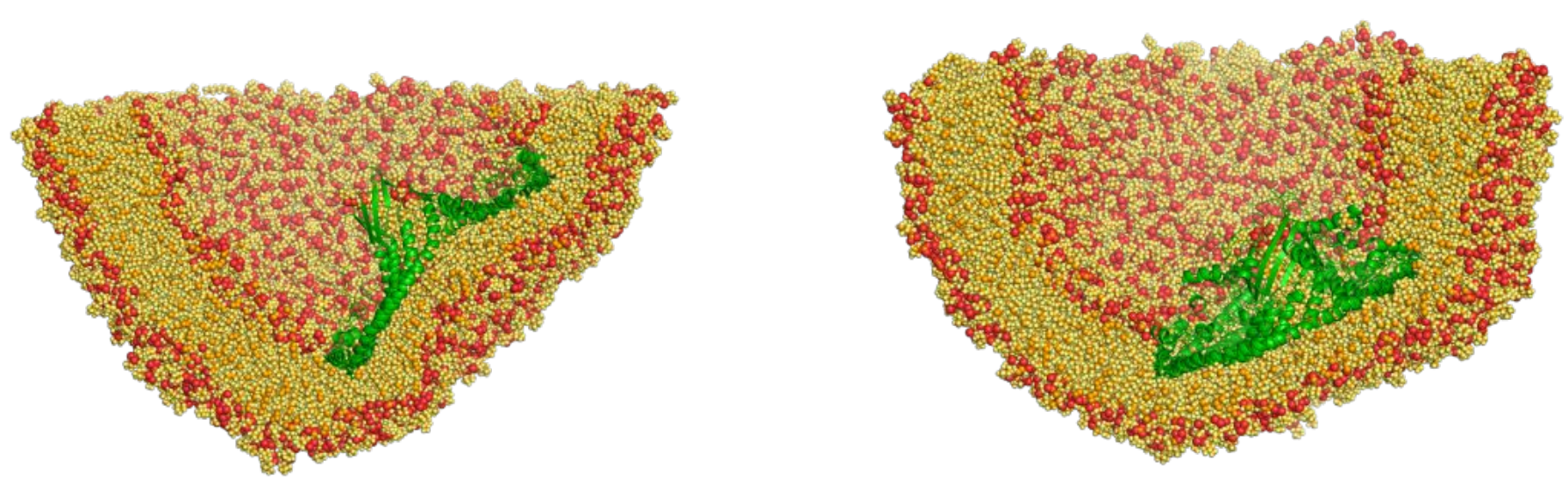

Fig. S2. Replicate runs of the nonvertebrate caveolin 8S complexes, 9DNO left and 9DN1 right. The radius of (negative) curvature of the outer leaflet is ~ 11.2 nm on the left and 11.5 nm on the right.

c) In all runs lipids were added inside the β-barrel to stabilize it. To check the possibility that this may have affected the results, we ran the POPC/cholesterol system without added lipids. This system produced results indistinguishable from the original run. One cholesterol molecule entered the barrel.

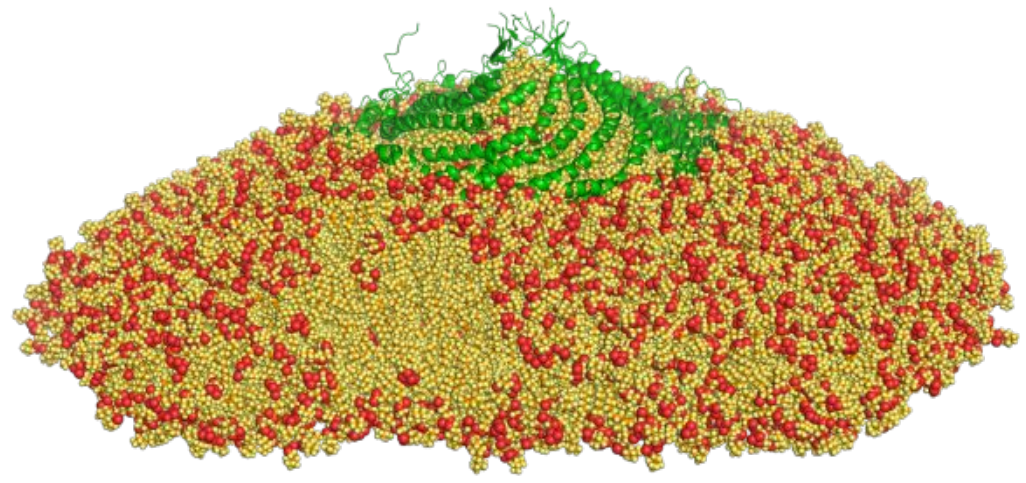

Fig. S3. 8S in POPC/cholesterol without added lipids in the barrel. The radius of curvature of the outer leaflet is ~ 20.0 nm.

d) All simulations were run at 310 K. To check the effect of temperature, we ran the POPC system at 303 K. Again, the result was indistinguishable. The change occurred slightly more slowly than at 310 K.

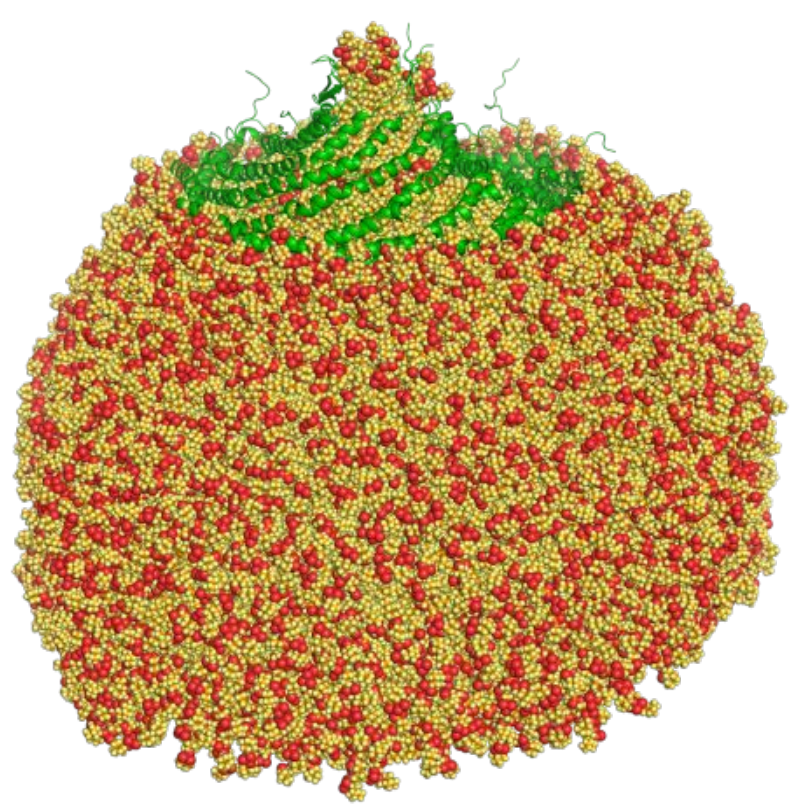

Fig. S4. 8S in POPC at 303 K. The radius of curvature of the outer leaflet is ~ 11.4 nm.

e) All simulations used the charmm36m FF for the protein and c36 for the lipids. To check the effect of the force field, we ran the POPC system using Amber 19SB for the protein and Amber21 for the lipids. Very similar results were obtained.

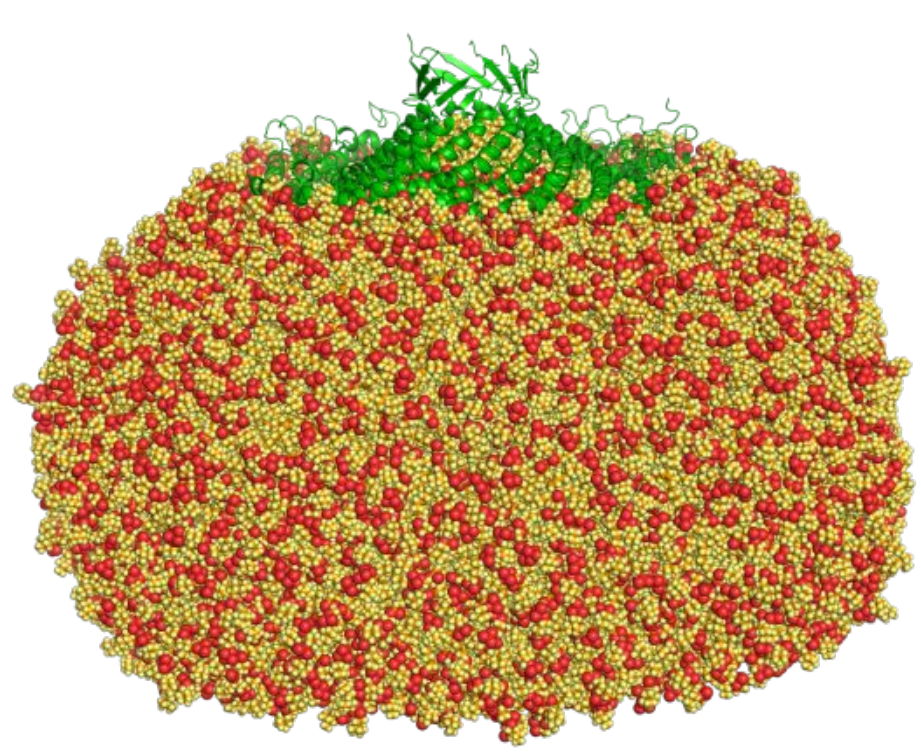

Fig. S5. 8S in POPC using Amber force fields. The radius of curvature of the outer leaflet is ~ 11.4 nm.

f) Most simulations used the semi-isotropic barostat. In the membrane only system this resulted in a significant change in the aspect ratio of the unit cell. In contrast, the isotropic barostat ran without issues. To check the effect of the barostat on membrane curvature, several systems were also run with the isotropic barostat. The 9DN0 replicate is shown above. POPC and POPC/cholesterol are shown in Fig. S6. The results were very similar.

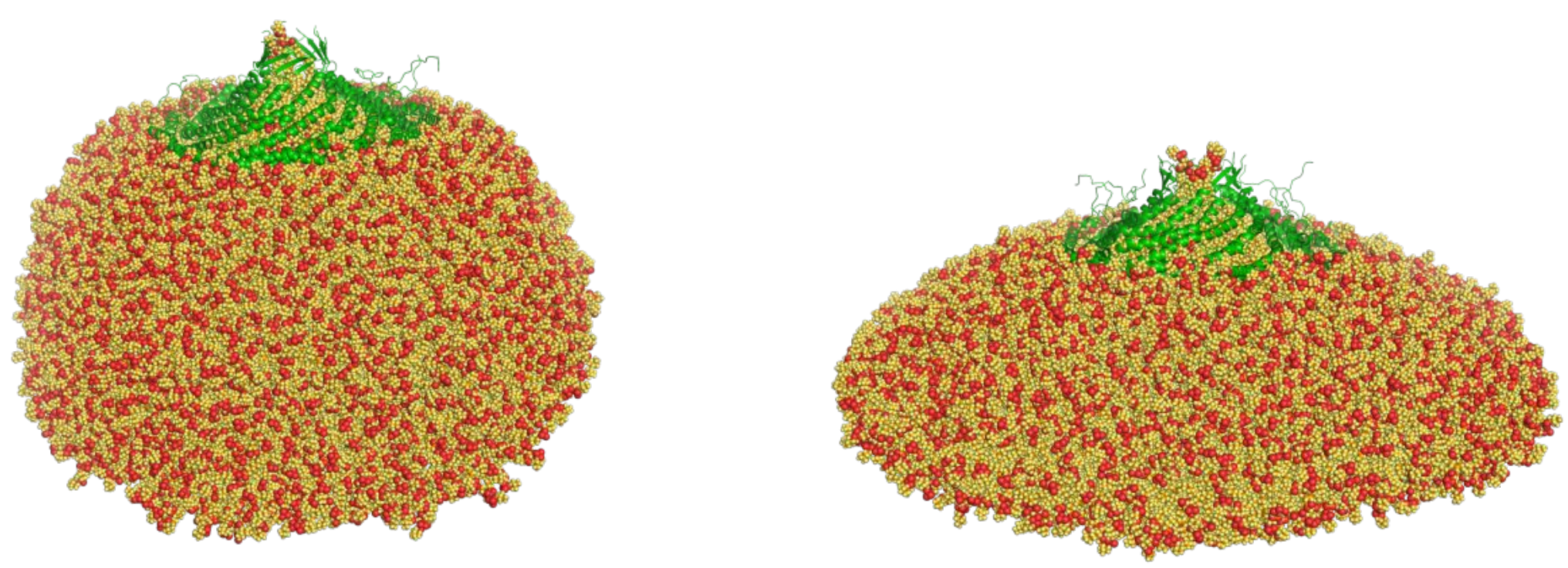

Fig. S6. 8S in POPC (left) and POPC/chol (right) using the isotropic barostat. The radius of curvature of the outer leaflet is ~ 12.5 nm on the left and 20.5 nm on the right.

g) To identify conditions under which the 8S complex remains flat, we ran a simulation in explicit n-hexane. The structure exhibited distortions, presumably due to the strong interactions between charged residues. The barrel half-dissolved, but did not rise as in the aqueous simulations.

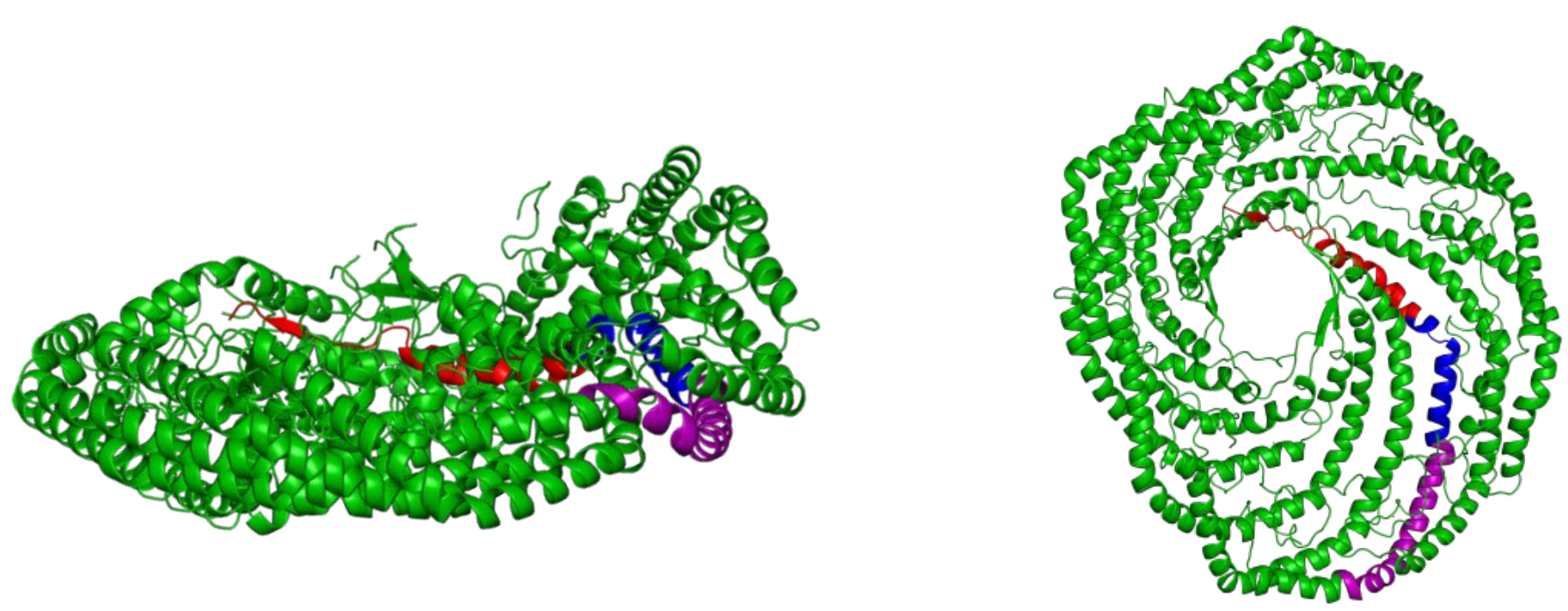

Fig. S7. Cav1 8S in explicit n-hexane, side and top views. One chain is colored according to domain.

h) The mutants P132L and S80E and cav2 are experimentally known to not form 8S complexes [1–3]. Nevertheless, we ran all three with the same protocol as cav1 and cav3 (a homology model using swissmodel was built for cav2). The P132L mutant gave very similar results to wild type (although in implicit solvent simulations, it remained flat). The S80E mutant also gave similar results to wild type. Cav2 remained flat and generated slightly negative curvature. Presumably, the 2 μs time scale is too short to reveal the thermodynamic instability of these complexes.

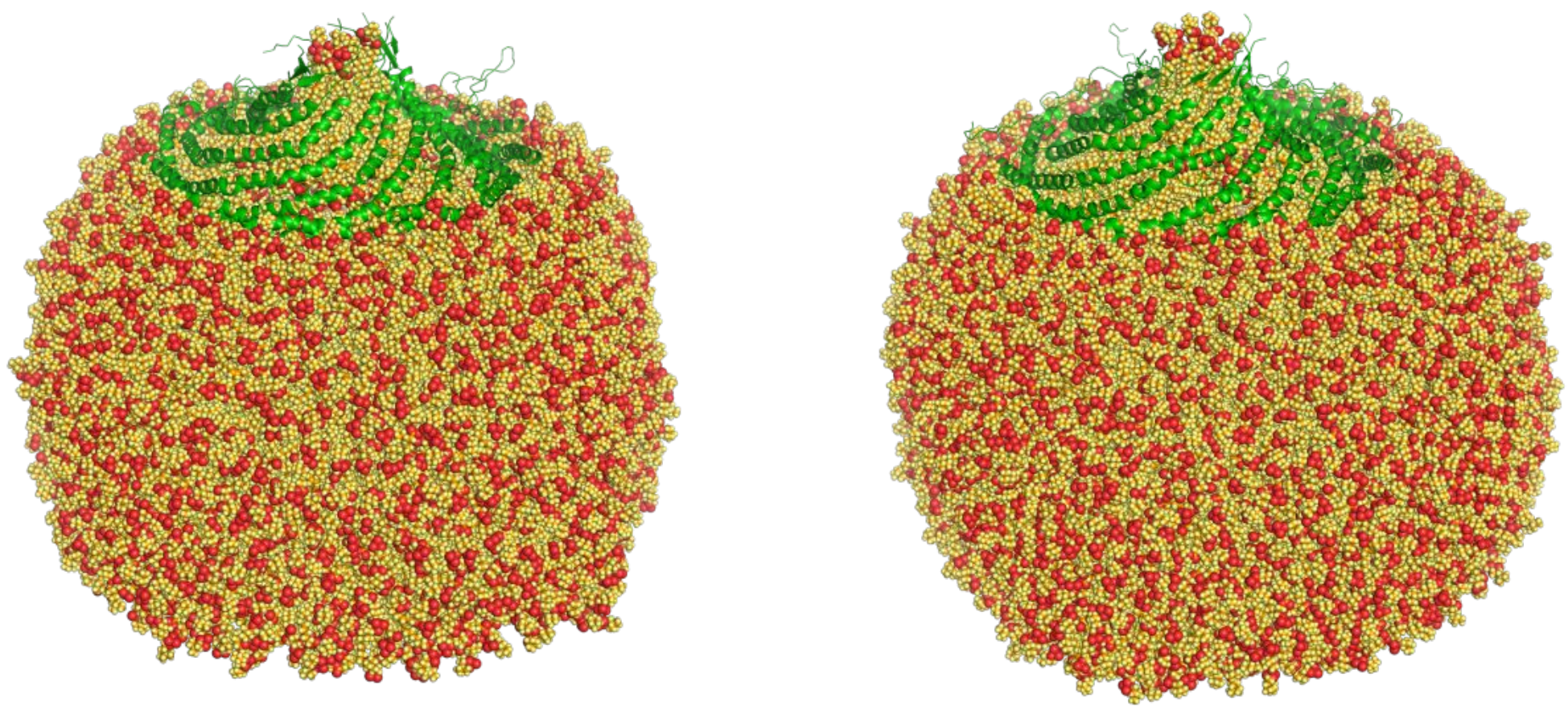

Fig. S8. P132L (left) and S80E (right) in POPC. The radius of curvature of the outer leaflet is ~ 10.9 nm on the left and 11.1 nm on the right.

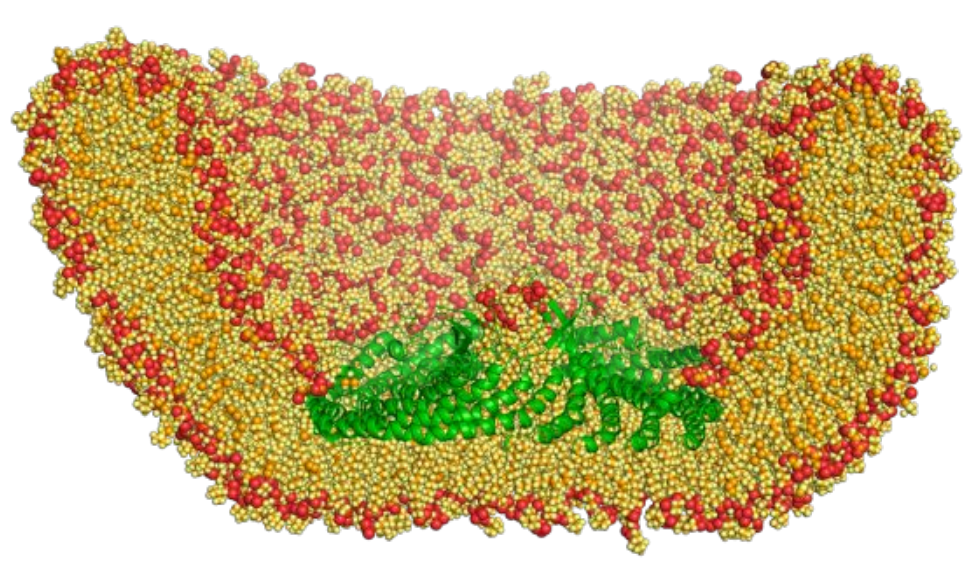

Fig. S9. Homology model of cav2 in POPC. The radius of (negative) curvature of the outer leaflet is ~ 11.6 nm.

i) Cav1 palmitoylated at Cys133 and Cys156 was run in POPC and gave very similar results to the unpalmitoylated system. A model for full-length cav1 was constructed based on the AF2 prediction. That also gave very similar results to the truncated complex.

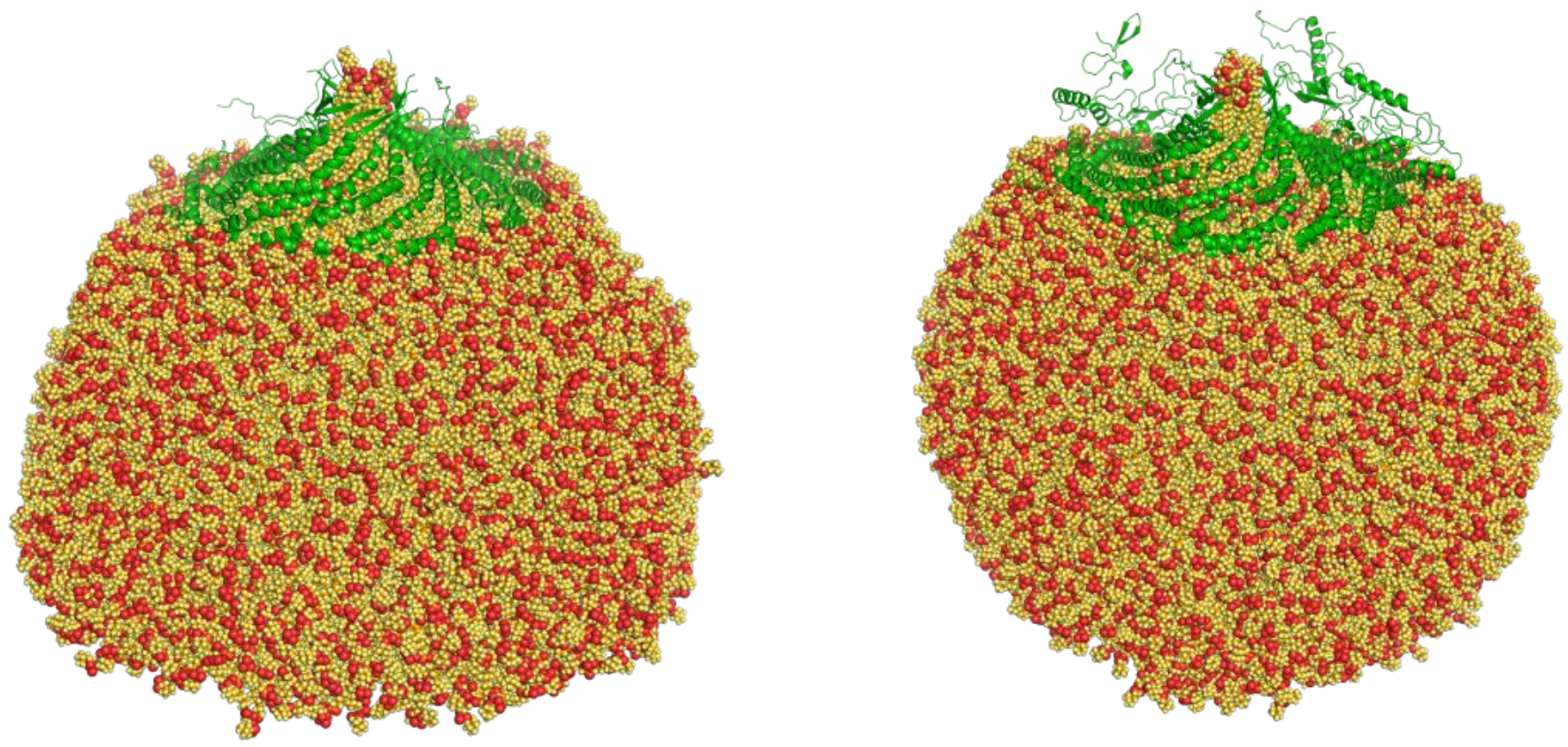

Fig. S10. Palmitoylated (left) and full-length cav1 (right) in POPC after 2 μs of simulation. The radius of curvature of the outer leaflet is ~ 11.2 nm on the left and 10.8 nm on the right.

References

(1) Li, S.; Galbiati, F.; Volonte', D.; Sargiacomo, M.; Engelman, J. A.; Das, K.; Scherer, P. E.; Lisanti, M. P. Mutational Analysis of Caveolin-Induced Vesicle Formation. Expression of Caveolin-1 Recruits Caveolin-2 to Caveolae Membranes. *FEBS Lett.* **1998**, *434* (1–2), 127–134. https://doi.org/10.1016/S0014-5793(98)00945-4.

(2) Hayer, A.; Stoeber, M.; Bissig, C.; Helenius, A. Biogenesis of Caveolae: Stepwise Assembly of Large Caveolin and Cavin Complexes. *Traffic* **2010**, *11* (3), 361–382. https://doi.org/10.1111/j.1600-0854.2009.01023.x.

(3) Kenworthy, A. K. The Building Blocks of Caveolae Revealed: Caveolins Finally Take Center Stage. *Biochem. Soc. Trans.* **2023**, *51* (2), 855–869. https://doi.org/10.1042/BST20221298.
